# Exploring the potential immunomodulatory effects of gallic acid on milk phagocytes in bovine mastitis caused by *Staphylococcus aureus*

**DOI:** 10.3389/fvets.2023.1255058

**Published:** 2023-09-15

**Authors:** Raktham Mektrirat, Phongsakorn Chuammitri, Dussaniya Navathong, Thofun Khumma, Anyaphat Srithanasuwan, Witaya Suriyasathaporn

**Affiliations:** ^1^Department of Veterinary Biosciences and Public Health, Faculty of Veterinary Medicine, Chiang Mai University, Chiang Mai, Thailand; ^2^Center of Excellence in Pharmaceutical Nanotechnology, Chiang Mai University, Chiang Mai, Thailand; ^3^Research Center of Producing and Development of Products and Innovations for Animal Health and Production, Chiang Mai University, Chiang Mai, Thailand; ^4^Doctoral Program in Veterinary Science, Faculty of Veterinary Medicine, Chiang Mai University, Chiang Mai, Thailand; ^5^Department of Animal Sciences, Wageningen University, Wageningen, Netherlands; ^6^Department of Food Animal Clinic, Faculty of Veterinary Medicine, Chiang Mai University, Chiang Mai, Thailand

**Keywords:** gallic acid, milk phagocyte, *Staphylococcus aureus*, immunomodulation, anti-inflammatory, apoptosis, bovine mastitis

## Abstract

Bovine mastitis caused by *Staphylococcus aureus* may exacerbate by resulting in significant economic losses and impacting milk quality. To date, the use of gallic acid, a phenolic compound naturally occurring in various plants, holds promise due to its potent anti-oxidant and anti-inflammatory effects in many pieces of literature, thus, making it a subject of interest in bovine innate immunity research. Here we used gallic acid to assess its potential immunomodulation on milk phagocytes *in vitro* challenges with mastitis-causing bacteria. Our findings indicated that cells exposed to gallic acid showed no harm to cell viability but might maintain the longevity of cells during the bacterial infection. Gallic acid-treated cells displayed reduced cell migration, phagocytosis, and bacterial killing ability, while showing an increase in ROS production, all of which are undoubtedly linked to the intracellular killing abilities of the cells. Nonetheless, the extracellular structure called neutrophil extracellular traps (NETs) was significantly released after receiving gallic acid, representing extracellular killing. We also reported that gallic acid neutralizes inflammation by regulating specific pro-inflammatory genes (*IL1B*, *IL6*, *TNF*) and ROS-generating genes (*CYBA*, *LAMP1*, *RAC1*), subsequently preventing tissue damage. Regarding apoptosis-related genes and proteins, the increased production of caspase-3 and Bcl-2 family proteins could potentially promote the longevity of cells, implicated in the mechanism of combating bacterial invasion during udder inflammation and infection. The novel role of gallic acid on milk phagocytes highlights its potential immunomodulatory properties and contributes to our understanding of its effects on bacterial-host interactions, and provides valuable molecular insights.

## Introduction

Bovine mastitis is a prevalent and economically consequential disease in dairy cattle, characterized by mammary gland inflammation. *Staphylococcus aureus*, a notable pathogenic microorganism, manifests various virulent traits that facilitate its persistence and sustenance within the udder, leading to persistent infections (1). *S. aureus* causes clinical and subclinical mastitis by infecting the mammary gland, reducing milk yield and increasing farmers’ financial burden. In some cases, culling chronically infected cows infected with *S. aureus* may be necessary to prevent the spread of infection (2). Implementing efficacious control measures, such as rigorous hygiene protocols, and adopting alternative control strategies, such as using herbal remedies as plausible therapeutic interventions, may mitigate the incidence of bovine mastitis caused by *S. aureus*. The use of herbal remedies with antimicrobial properties, along with boosting udder immunity at the localized level, maybe a viable and sustainable approach to controlling and treating bovine mastitis (3). Numerous herbs contain compounds, including gallic acid and anacardic acid, with antimicrobial and anti-inflammatory characteristics. These properties may aid in preventing the growth of mastitis-causing pathogens and alleviate mammary gland inflammation.

Gallic acid (GA) and its derivatives are prevalent in numerous plant-based foods and beverages (4, 5). They are widely used in industry and have intriguing potential as animal feed additives (6). Due to its potent removal of free radicals, gallic acid is an effective anti-oxidant that participates in many other beneficial biological processes (4, 7, 8). Gallic acid can modulate the immune response, and this possesses the potential to assist in natural defense against bacterial infections (7, 9). Consequently, gallic acid could serve as a viable alternative to antibiotics in bovine mastitis treatment, contributing to the reduction of antimicrobial usage in the dairy industry and preventing the development of antibiotic resistance, a serious public health concern.

With its anti-inflammatory and immunomodulatory qualities, gallic acid has recently received attention for its immunostimulant properties, which contribute to its potential immunological modulation effects via the control of cytokines and histamine production (10, 11). The transcription factor NF-κB has attracted extensive research attention from numerous investigators due to its function in cell signaling concerning inflammatory mediators and anti-oxidants that manage cell death and senescence. Several reports have pointed out the usefulness of gallic acid in inhibiting this element (12–16). Because gallic acid has immune-enhancing properties, it is a promising therapeutic candidate. Gallic acid has been studied over the past decade for its effects on human and animal innate immune cells, primarily macrophages and neutrophils, and the results show promise for potential therapeutic use (11, 17, 18). Unfortunately, there is a lack of direct information regarding the effects of gallic acid on bovine polymorphonuclear leukocytes (PMNs). Several studies have demonstrated that gallic acid can precisely modulate the activity of the innate immune system cells. Notably, it modulates vital cellular functions such as phagocytosis, bacterial killing, degranulation, ROS production, cell migration, and NET release (19, 20). However, the exact mechanisms of how gallic acid acts on the innate immune system remain unclear.

Despite the scarcity of published studies on the effects of gallic acid on bovine milk phagocytes and their immune response to mastitis-causing bacteria, there is a promising avenue for additional research. This study aimed to assess the effects of gallic acid on milk phagocyte activities during *in vitro Staphylococcus aureus* challenge, shedding light on another aspect of gallic acid’s potential to modify cellular longevity during bacterial infections that trigger mastitis. By investigating these facets, we can uncover valuable insights into the potential benefits of gallic acid in modulating the immune response and combating mastitis-causing bacteria.

## Materials and methods

### Collection of milk samples and isolation of milk phagocytes

The dairy cow milk samples were obtained from smallholder dairy herds in Mae-Wang Dairy Cooperative, Chiang Mai, Thailand. In this study, crossbred Holstein-Friesians (*Bos taurus*) aged between 3.5–7 years and weighing 350–400 kg in their second to fourth lactation periods, housed in tie-stall barns with concrete floors, were recruited for animal subjects. Using bucket-style milking equipment, lactating cows were milked twice a day. Information on cow characteristics, herd/milking management, and milking equipment has been collected. Each of the three herds had 24, 29, and 37 milking cows, with average daily milk production of 6.5, 8.7, and 7.2 kg, respectively. All quarters were screened for subclinical mastitis using the California mastitis test (CMT), and milk samples were collected aseptically from quarters with a CMT of 2 so that bacteria could be later identified. In clinical cases requiring treatment, the local veterinarian will administer antibiotics. The research protocol involving animals underwent a thorough review and received approval from the Animal Care and Use Committee (FVM-ACUC), Ref. No. S27/2564, at the Faculty of Veterinary Medicine, Chiang Mai University. Sixteen milk samples were collected from quarters that met the specified requirements. Each milk sample, amounting to two tubes of 15 mL each, was promptly used to isolate milk phagocytes following established procedures (3).

Briefly, the milk from each quarter was subjected to centrifugation, and the cream and whey layers were discarded. The resulting cell pellet was washed twice using cold Hank’s Balanced Salt Solution (HBSS) and resuspended in cold RPMI-1640 medium supplemented with 1% fetal bovine serum (FBS). The isolated milk phagocytes from each quarter were divided into the control group (HBSS) and the gallic acid treatment group (GA). The viability of the isolated cells was determined using trypan blue dye exclusion. To assess the differential cell population from the isolation process, a cytospin preparation slide was used to review milk cells. Neutrophils were the most abundant cell type. Finally, the cell density was adjusted to approximately 3 × 10^6^ cells per mL.

### Bacterial growth conditions, fluorescent labeling, and opsonization

The bacterial strain *Staphylococcus aureus* (*S. aureus*) ATCC 25923 was utilized throughout the experiment. Before its use in the experimental procedures, a portion of the frozen bacterial stock was thawed and inoculated onto Tryptic soy agar (TSA, HIMEDIA, Mumbai, India) plates supplemented with 5% bovine blood. The plates were then incubated overnight at 37°C for bacterial growth. Subsequently, the bacterial population was adjusted to approximately 10^8^ colony-forming units per milliliter (CFU/mL) prior to its use in the experiment. Live *S. aureus* cells were subjected to heat-killing and fluorescent labeling, following the methods outlined by Disbanchong et al. (3). The fluorescently labeled *S. aureus* cells, at a concentration of 10^7^ CFU/mL, were opsonized with 10% heat-inactivated autologous bovine serum for 20 min at 37°C before being utilized in the phagocytosis assay.

### Preparation of gallic acid

Gallic acid (GA), also known as 3,4,5-trihydroxy benzoic acid, with a molecular weight of 170.12 g/mol, was procured from Sigma-Aldrich (Cat. No. G7384). The stock gallic acid solution (1 mM) and the working solution (50 μM) were prepared according to the following procedure. In brief, the gallic acid powder was dissolved in filter-sterilized water and dimethyl sulfoxide (DMSO) to create the stock solution. The stock solution was then diluted 1:20 with HBSS to generate the working solution. All solutions were prepared freshly on the day of the experiment and stored at room temperature until employed.

### Determination of *Staphylococcus aureus* viability

The assessment of the anti-bacterial activity of gallic acid through *in vitro* assays was conducted following a previously described method with modifications (3). The procedure involved washing an aliquot of *S. aureus*, which was subsequently diluted to a concentration of 0.5 McFarland in Tryptic soy broth (TSB). The agar gel diffusion assay was performed utilizing sterile Whatman filter paper disks with a diameter of 6 mm. These disks were immersed in different concentrations of gallic acid (10, 50, 100, 500 μM, and 1 mM), 80 μM H_2_O_2_, 1× HBSS as a control for 1 min, and a Streptomycin disc (Oxoid Limited, Hampshire, United Kingdom). The prepared disks were carefully positioned onto spread plates inoculated with *S. aureus*, ensuring complete and uniform contact with the bacteria. Subsequently, the plates were incubated at 37°C for 16 h. Images were captured for subsequent analysis using the GelMax Imager (Ultra-Violet Products, Cambridge, United Kingdom).

To quantify the bacterial colonies following treatment with gallic acid, a modified protocol for bacterial enumeration using the drop plate method was employed, based on the methodology described by Thomas et al. (21). The procedure involved the serial dilution of bacterial samples, spanning five dilutions (ranging from 10^−1^ to 10^−5^), followed by drop plating on agar in a 6 × 5 format. An aliquot of *S. aureus* at a concentration of 0.5 McFarland (100) was treated with various concentrations of gallic acid (10, 50, 100, 500 μM, and 1 mM), as well as Streptomycin (Gibco) for a duration of 1 h. Subsequently, 250 μL of the sample (consisting of 125 μL each of *S. aureus* and the respective concentration of gallic acid) was loaded into the first well of each row in a 96-well plate. Ten-fold serial dilutions (ranging from 10^−1^ to 10^−5^) were prepared by transferring 20 μL from each selected dilution into 180 μL of the medium. Next, 20 μL droplets from the chosen dilutions were plated onto an agar medium. The droplets on the plates were allowed to dry for approximately 3–4 min in a laminar airflow cabinet before being transferred to an incubator. After 16 h of incubation at 37°C, the developed colonies (ranging in size from 0.5 mm to 1 mm) were enumerated. The colony growth pattern at varying dilutions was categorized as too many to count (TMTC) or countable/acceptable (6–60 colonies). Upon recording the dilution level that yielded acceptable colonies and the colony-forming units (CFU) per sector, the CFU per 100 μL was calculated as *n* × 5 (where *n* represents the number of colonies in the 20 μL sample application area). The CFU/mL of the original 10^0^ stock was determined by (*n* × 5) × 10^(*d* + 1)^, where *d* represents the dilution level that resulted in countable colonies (21).

### *In vitro* milk phagocyte cytotoxicity assessment

A total of 1 × 10^5^ isolated milk phagocytes were seeded in duplicate into a 96-well flat-bottom plate and subsequently incubated with either HBSS as control or various dilutions of gallic acid (12, 25, 50, 100, and 200 μM) in RPMI-1640 medium. The plate was incubated at 37°C with 5% CO_2_ for 1 h. Following incubation, the plate was centrifuged at 1,200 rpm for 3 min, and the supernatant was discarded. Subsequently, all wells were treated with 2 μg/mL of MTT in RPMI-1640 medium and incubated for 90 min. After centrifugation, the medium containing non-metabolized MTT was aspirated, and 150 μL of dimethyl sulfoxide (DMSO) was added to solubilize the formazan crystals formed by viable cells (3). To indicate the baseline color in the assay, the MTT solution (Blank) was added. The absorbance (OD) was then measured at a wavelength of 570 nm using a microplate reader (Anthos Labtec Instruments, Wals, Austria). The percentage of cell viability was quantified using the following equation:


$$\%\;\mathrm{viable}\;\mathrm{cells}=\;\frac{\left({OD}_{\mathrm{gallic}\;\mathrm{acid}}-{OD}_{\mathrm{blank}}\right)}{\left({OD}_{\mathrm{control}}-{OD}_{\mathrm{blank}}\right)}\times 100$$

The percentage of milk phagocyte cell death following treatment with various concentrations of gallic acid was calculated by subtracting the percentage viability from 100. A dose–response curve was generated, with gallic acid concentration represented on the *x*-axis and the percentage of milk phagocyte cell death on the *y*-axis.

### Quantification of intracellular reactive oxygen species (ROS)

For unraveling the reactive oxygen species (ROS) molecules, milk phagocytes were seeded at a density of 1 × 10^5^ cells per well in duplicate 96-well flat tissue culture plates. Treatment followed with either 50 μM gallic acid (GA) for 30 min at 37°C with 5% CO_2_ or HBSS as the control. Post-treatment, cells were washed, centrifuged, and loaded with 10 μM of 2′,7′-dichlorofluorescein diacetate (H_2_DCF-DA, Invitrogen), a fluorescent probe for H_2_O_2_ staining. After a 15-min incubation in the dark and a subsequent cold wash with HBSS, ROS-containing cells were detected using a DxFLEX Flow Cytometer (Beckman Coulter, Brea, CA, United States). FlowJo 10 software facilitated data analysis (Treestar, Ashland, OR, United States).

### Assessment of phagocytosis

The phagocytic activity of *S. aureus* was evaluated using flow cytometry. Briefly, milk phagocytes (1 × 10^5^ cells) were treated with either gallic acid or HBSS for 30 min. Subsequently, the treated cells were mixed with opsonized fluorescently labeled *S. aureus* [at a multiplicity of infection (MOI) of 10] in duplicate wells of a 96-well flat-bottom cell culture plate. To facilitate the uptake of *S. aureus*, the cell mixture was subjected to centrifugation at 1,200 rpm for 3 min, allowing the milk phagocytes to internalize the bacteria for 45 min at 37°C with 5% CO_2_. Following incubation, the cells were extensively washed with ice-cold HBSS, and sample acquisitions were performed using a flow cytometer.

### Under-agarose cell migration assay

We employed a modified version of established protocols to investigate the direct migration of milk phagocytes toward viable bacteria, as previously described (22, 23). Initially, 0.5 g of agarose powder (Bio Basic Inc., Ontario, Canada) was dissolved in 10 mL of sterile distilled water and heated to boiling to ensure complete dissolution. After cooling to 48°C in a water bath, the agarose solution was mixed with a prewarmed cell culture medium consisting of 20 mL of RPMI 1640 supplemented with 5% FBS and 10 mL HBSS, resulting in a final volume of 40 mL (referred to as Agarose medium). Each 90 × 15-mm petri dish (SPL Life Sciences, Gyeonggi-do, Korea) was filled with 7 mL agarose medium and solidified at RT for 15 min. Using a stainless-steel hole punch, six series of seven wells, each measuring 3.5 mm in diameter, were created in the agarose gel plates to accommodate the desired configuration. Agarose plugs were then removed from the wells using a pointed-tip tweezer. The gel plates were equilibrated at 37°C with 5% CO_2_ for 30 min. Milk phagocytes (1 × 10^5^ cells) were treated with 50 μM gallic acid or HBSS for 30 min. Subsequently, 20 μL of the treated milk phagocytes (containing approximately 6,000 cells) or 20 μL of live *S. aureus* (containing approximately 6 × 10^5^ bacteria) were loaded into the corresponding wells. The Petri dishes were incubated at 37°C with 5% CO_2_ for 2 h. After incubation, the cells were fixed *in situ* by adding 6 mL of methanol (Sigma-Aldrich) to the agarose gel for 5 min. Following fixation, the dishes were stained with DipQuick (RVL Supply, Bangkok, Thailand), washed twice with distilled H_2_O, and air-dried at room temperature. Migration patterns, specifically the areas between the milk phagocyte-containing wells and bacteria-containing wells, were captured at magnifications of 4× and 10× using an Olympus BX53 Upright Microscope (Olympus Corporation, Tokyo, Japan). Migrated cells were quantified and analyzed using ImageJ v1.52a software with a cell counter plug-in [National Institutes of Health (NIH), Bethesda, MD, United States].

### Evaluation of bacterial killing by MTT assay and spot dilution assay

The bactericidal capacity of milk phagocytes was assessed using a semi-quantitative MTT assay to determine the percentage of bacterial viability. A qualitative spot plate assay was also performed for colony scoring after the killing assay, following a previously described method with some modifications (3). *S. aureus* was freshly cultured, as outlined in the previous section. The live bacteria were opsonized with autologous bovine serum to obtain a final concentration of 1 × 10^7^ CFU/mL. In duplicate wells of a 96-well plate, milk phagocytes (1 × 10^5^ cells) were loaded and treated with either 50 μM gallic acid or HBSS for 30 min. Subsequently, the opsonized bacteria were added at a 1:10 ratio to the wells containing the treated milk phagocytes. The plate was centrifuged at 1,200 rpm for 3 min and incubated for 45 min. After incubation, the plate was centrifuged again to remove non-ingested bacteria. A hypotonic solution (diH_2_O) was used to release the internalized bacteria from the milk phagocytes, followed by a 5-min incubation at RT. A portion of the cell lysates was collected for spot dilution assays, while the remaining cell lysates were supplemented with tryptic soy broth (TSB) containing 2 μg/mL MTT. The plate was then incubated for a total of 90 min at 37°C to allow the MTT-insoluble formazan to form colored crystals. Subsequently, the formazan was solubilized by adding DMSO, and colorimetric detection was performed at a wavelength of 570 nm. In each experiment, an OD measurement was obtained from the MTT solution (Blank) to confirm the absence of live bacteria. The percentage of bacterial killing was calculated using the following formula:


$$\%\;\mathrm{of}\;\mathrm{killing}=100-\left[\left({OD}_{\mathrm{sample}}-{OD}_{\mathrm{blank}}\right)\times 100\right]$$

Spot dilution assays were conducted using a 2 μL aliquot of lysed milk phagocytes obtained from the previous steps in the MTT assay. A series of 10-fold dilutions (2 μL each of 10^−1^ to 10^−6^) was carefully spotted onto Tryptic soy agar (TSA) plates and allowed to grow overnight (16 h) at 37°C. Images were captured using the GelMax Imager to facilitate a comparison of colony sizes. The number of colonies was determined using the equation described in section “Determination of *Staphylococcus aureus* viability.”

### Quantification and visualization of neutrophil extracellular traps (NETs)

To quantify and visualize the release of neutrophil extracellular traps (NETs) from isolated milk phagocytes, duplicate wells of a 96-well plate were seeded with 1 × 10^5^ cells per well. The cells were stimulated with either HBSS as a control, 50 μM gallic acid, or 100 nM phorbol 12-myristate 13-acetate (PMA), a pharmacological inducer of NETs. Additionally, all wells were supplemented with HBSS containing Ca^2+^ and Mg^2+^ before incubation at 37°C with 5% CO_2_ for 150 min. Following activation, the plates were centrifuged at 1,200 rpm for 3 min, and the supernatant was removed. Ice-cold RPMI 1640 media was added to each well, gently mixed by pipetting, and then centrifuged. The supernatant containing extracellular DNA was transferred to new plates. NET-DNA was quantified using a fluorescent dye, Hoechst 33342 (Invitrogen), at a concentration of 5 mg/mL (3). The fluorescence emitted by the stained NETs was measured using a Synergy™ HT Multi-Detection Microplate Reader with an excitation wavelength of 360 nm and an emission wavelength of 470 nm. The fluorescence intensity was recorded as relative fluorescence units (RFU).

NET structures were confirmed through fluorescence microscopy analysis by staining the NET structures derived from HBSS or gallic acid treatments using DAPI/propidium iodide for DNA and H_2_DCF-DA for reactive oxygen species (ROS), following a previously established method (3). Briefly, isolated cells (1 × 10^5^ cells) were seeded into 8-well chamber slides (SPL Life Sciences, Korea) and stimulated with HBSS or 50 μM gallic acid. Ca^2+^ and Mg^2+^ were supplemented in all wells. The cells were stimulated for 180 min at 37°C with 5% CO_2_. After stimulation, all samples were rinsed with ice-cold PBS and fixed with 4% paraformaldehyde (PFA) for 15 min. The slides were then rinsed with ice-cold PBS and stained with a solution of 10 mg/mL propidium iodide (PI, Sigma-Aldrich) and a final concentration of 10 μM H_2_DCF-DA for 10 min in the dark, followed by rinsing. The chamber slides were disassembled, and a drop of ProLong Gold Antifade Mountant with DAPI (Invitrogen) was applied. Visualization and image capture was performed using an Axio Scope A1 Fluorescence Microscope (Carl Zeiss, Thornwood, NY, United States).

### Quantitative real-time PCR (qPCR)

To investigate the impact of gallic acid on gene expressions during exposure to *S. aureus*, milk phagocytes (2 × 10^6^ cells) were placed in microcentrifuge tubes and stimulated for 2 h with a combination of 50 μM gallic acid and live *S. aureus* (MOI of 10), or HBSS and live *S. aureus* as control. Subsequently, cells were washed with HBSS, collected through centrifugation, and the resulting pellets were resuspended in RNA*later* (Invitrogen) following the manufacturer’s guidelines to preserve RNA integrity. As instructed, the preserved RNAs were then extracted using the RNAzol® RT (Sigma-Aldrich). For cDNA synthesis, 2 μg of total RNA was utilized with the Tetro cDNA Synthesis Kit (Bioline, Taunton, MA, United States), and 100 ng of the resulting cDNA samples from milk phagocytes were subjected to quantitative analysis in triplicate. The mRNA transcripts of *IL1B*, *IL6*, *TNF*, *CXCL8*, *CYBA*, *LAMP1*, *RAC1*, *BAX*, *BCL2*, *BCL2L1*, *CASP3*, *CASP9*, *CFLAR*, *FAS*, *MIR29B-2*, *MIR146A*, *MIR155*, *MIR223*, *MIR16B*, and *ACTB* were quantified using real-time PCR with the SensiFAST SYBR Hi-ROX Kit (Bioline) on an ABI Prism 7,300 real-time PCR instrument (Applied Biosystems, Thermo Fisher Scientific). The selection of *ACTB* or *MIR16B* as the endogenous control allowed for normalizing gene expression levels (3, 24). The 2^−ΔCt^ method was employed to calculate the fold-change (Log2FC) in gene expression, with the Log2FC of the control group set as zero. Mean values ± SEM were reported. The primer sequences employed in this study were obtainedfrom the previously mentioned work (3, 24). Nonetheless, *CXCL8*, *BAX*, and *CASP9* were newly designed for this investigation. The primer sequences for *CXCL8* were designated as follows: *CXCL8*-Forward sequence was 5′-TCTCTGCAGCTCTGTGTGAAG-3′, and *CXCL8*-Reverse sequence was 5′-TTCCTTGGGGTTTAGGCAGAC-3′. The primer sequences for *BAX* were denoted as *BAX*-Forward (5′-TCGCCCTTTTCTACTTTGCC-3′) and *BAX*-Reverse (5′-TCGAAGGAAGTCCAATGTCCAG-3′). Similarly, the forward primer, denoted as *CASP9*-Forward, had the sequence 5′-GAGTCAGGCCCTTCCTTTGTT-3′, while the reverse primer, *CASP9*-Reverse, had the sequence 5′-CGGCTTTGATGGGTCATCCT-3′. All newly designed primers were annealed at 60°C.

### Western blot

For the analysis of Caspase-3 and Bcl-2 in milk phagocytes incubated with gallic acid, total protein was extracted from 2 × 10^6^ cells treated with HBSS or 50 μM gallic acid in the presence of live *S. aureus* (MOI of 10) for 2 h. The cell pellets were lysed using RIPA lysis buffer (Sigma-Aldrich) supplemented with a protease inhibitor cocktail (Sigma-Aldrich). The protein concentration of the extracted samples was determined by Bradford protein assay (Bio-Rad, Hercules, CA, United States). After that, the protein samples (30 μg) were combined with 2× Laemmli sample buffer (Bio-Rad) containing β-mercaptoethanol and heated at 95°C for 5 min. Next, proteins were separated on 12% SDS-PAGE gels and transferred onto a nitrocellulose membrane of size 2 μm pores (Bio-Rad), utilizing a Trans - Blot® SD Semi-Dry Transfer Cell (BioRad). After blocking the membranes in Tris-buffered saline with 0.05% Tween (TBST) and BSA (BSA, Bio Basic, Markham, ON, Canada) for an hour, they were incubated with purified mouse anti-caspase 3 monoclonal antibody (clone 4-1-18, BioLegend) and purified mouse anti-Bcl2 monoclonal antibody (clone BCL/10C4, BioLegend) in a dilution of 1:1,000 at RT, for 2 h. Thereafter, the membranes underwent incubation with a secondary antibody labeled as HRP-conjugated goat anti-mouse IgG antibody (clone Poly4053, BioLegend) in a dilution of 1:3,000 for approximately 45 min at RT. After washing the membranes with TBST, the detection was performed using a DAB substrate (Bio Basic). The quantification of the targeted protein levels was conducted using Image Studio Lite software (LI-COR, Lincoln, Nebraska, United States), with β-actin (clone 2F1-1, anti-actin Antibody, BioLegend) employed as a loading control at a dilution of 1:3,000.

### Statistical analysis

In all, two separate experiments were conducted to procure the data. The Gaussian distribution of the data was then assessed using the Shapiro–Wilk normality test, which evaluates its normality. The unpaired Student’s *t*-test was applied to the data of most assays unless otherwise indicated. Only assays with more than two treatment groups were required, either the one-way ANOVA or the Kruskal-Wallis test, followed by Dunnett’s multiple comparisons as post-tests. The whole statistical analyses in the current work were performed by the built-in statistical features of the GraphPad Prism 9.0 software (GraphPad Software, San Diego, CA, United States). The statistical significance was calculated and set at *p* < 0.05. Data are present as mean values with standard error (mean ± SE). The heatmap and bubble plot was generated by using RStudio version 2023.06.0 Build 421, interfaced with R version 4.2.1 (2022-06-23 ucrt) with R packages; *ggplot2*, *gplots*, *viridis*, and *RColorBrewer*.

## Results

### Determination of *Staphylococcus aureus* viability indicated that bacteria may withstand treatment with various sublethal doses of gallic acid

**Figure 1:**
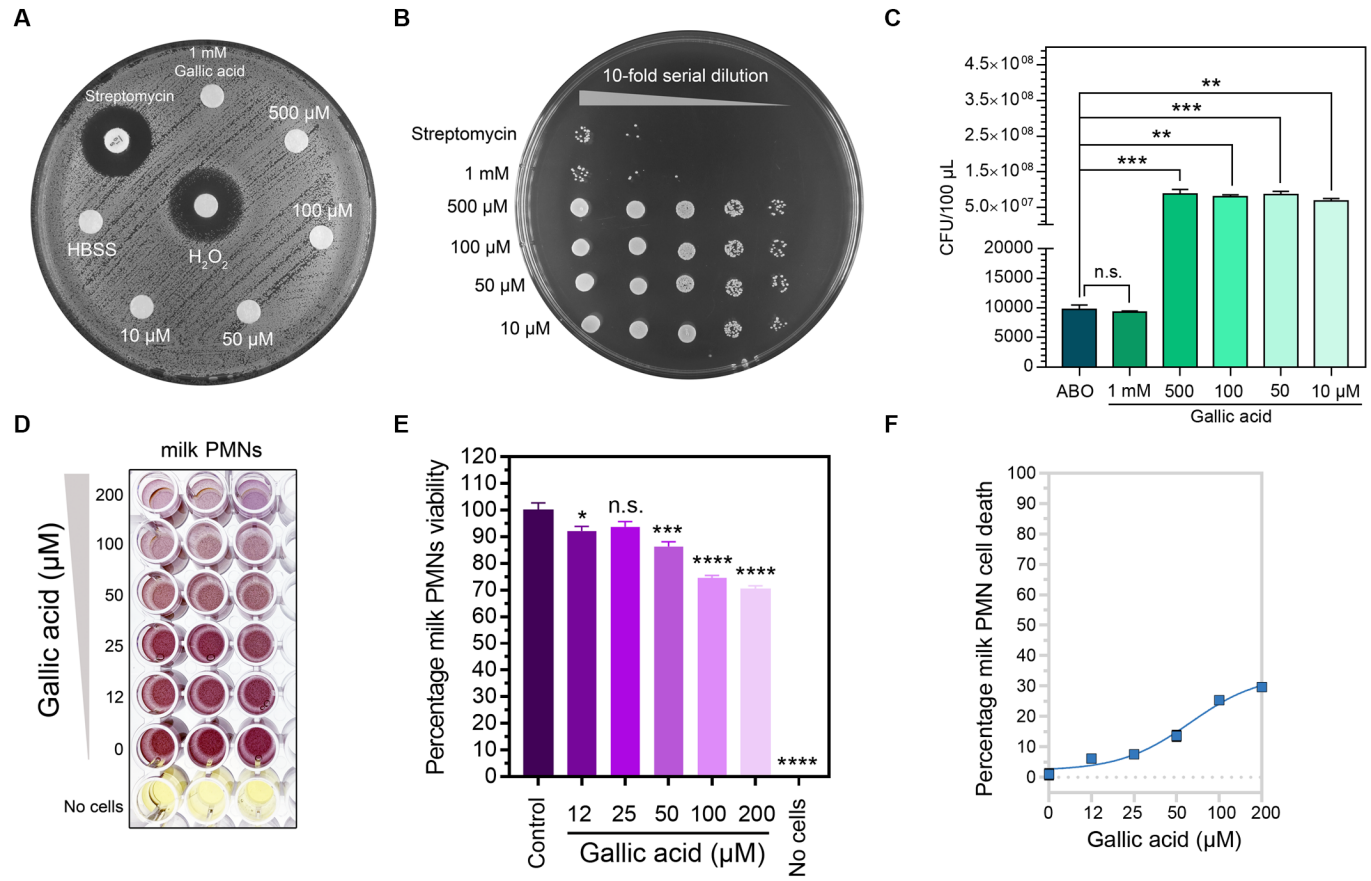
The anti-bacterial property of gallic acid on *Staphylococcus aureus* and safety of gallic acid in viability testing on milk phagocytes. **(A)** Results of disk diffusion assay. *Staphylococcus aureus* on an agar plate interacted with 1× HBSS, 10 μM, 50 μM, 100 μM, 500 μM, and 1 mM of gallic acid. The size of the clear zone indicates the effect of gallic acid or H_2_O_2_, or streptomycin on the inhibition of bacterial growth. No zone of growth inhibition is observed around all concentrations of gallic acid. **(B)** Growth of colonies of *Staphylococcus aureus* from drop plate method. Five dilutions (10^−1^ to 10^−5^) of the gallic acid and streptomycin treatment were plated and incubated for 16 h. **(C)** Enumeration of *Staphylococcus aureus* by drop plate method after treating bacteria with different concentrations of gallic acid and streptomycin (ABO). Data are from two separate experiments. **(D)** Purple formazan crystals formed by milk phagocytes under increasing concentrations of gallic acid or left untreated (0 μM) by MTT assay. **(E)** Cell viability was assessed by the MTT assay of milk phagocytes treated with different concentrations of gallic acid. The columns represent the mean ± SE (*n* = 3 per concentration). Means in each treatment differ significantly (*p* < 0.05) from that of the control based on one-way ANOVA and Dunnett’s multiple comparisons tests. **(F)** The dose–response curve plots the percentage of milk phagocyte cell death vs. gallic acid concentration resulting in a sigmoidal curve (*n* = 3 per concentration). Statistically significant *p* values are indicated in the graphs as follows: ****p < 0.0001, ***p < 0.001, **p < 0.01, and *p < 0.05 between groups, n.s. not significant.

Based on the experiment results, it was evident that gallic acid did not exhibit any direct bactericidal effect on *S. aureus* at any concentrations tested using the agar diffusion method. In contrast, the clear zones around the Streptomycin disc or H_2_O_2_ indicated that the bacteria could not survive on the agar (Figure 1A). Thus, we conclude that the bacterium is resistant to direct killing by gallic acid, irrespective of its concentration. To confirm the bactericidal effect of gallic acid, we conducted serial spot dilution assays by spotting bacterial colonies onto nutrient agar plates. The results revealed no discernible differences in the size of bacterial colonies among gallic acid-treated bacteria at concentrations of 10–500 μM except for the concentration at 1 mM (Figure 1B), compared with Streptomycin. These findings indicate that gallic acid may possess direct sublethal properties (at 1 mM) in its ability to kill bacteria (Figure 1C).

### The *in vitro* assessment of milk phagocyte cytotoxicity demonstrated that cell viability remained unaffected even after exposure to different concentrations of gallic acid

Our study aimed to assess if gallic acid had any toxicity toward milk phagocytes. In order to achieve our objective of determining cell survival rates, we supplemented a variety of concentrations of gallic acid (0–200 μM) to milk phagocytes. To detect cytotoxicity and assess the presence of live cells with functional mitochondria following exposure to gallic acid, the MTT assay was conducted (Figure 1D). Our results revealed that all concentrations (12–200 μM) of gallic acid tested gave a range of cell viability percentages between 70.28 and 92.18 (Figure 1E). The effects of gallic acid on cell toxicity can be understood by studying the dose–response curve and assessing the safety level of gallic acid that acted upon milk phagocytes (Figure 1F). When the gallic acid concentration was in the range of 12–200 μM, it was noticed that about 6.15%–29.72% of cells showed a response towards gallic acid. In contrast, less than half suffered from cell death, with an EC_50_ value of 50.29 μM. At a concentration of 50.29 μM or roughly 50 μM, there was a recorded percentage of approximately 14% of cell death. After considering these results, we decided to utilize an overall concentration of 50 μM throughout our research due to its ability to maintain cellular viability levels above and beyond 85% while keeping cell death numbers below 15% (Figure 1F).

### Flow cytometry-based quantification of intracellular ROS demonstrated a slight increase in the levels of ROS within milk phagocytes treated with gallic acid

**Figure 2:**
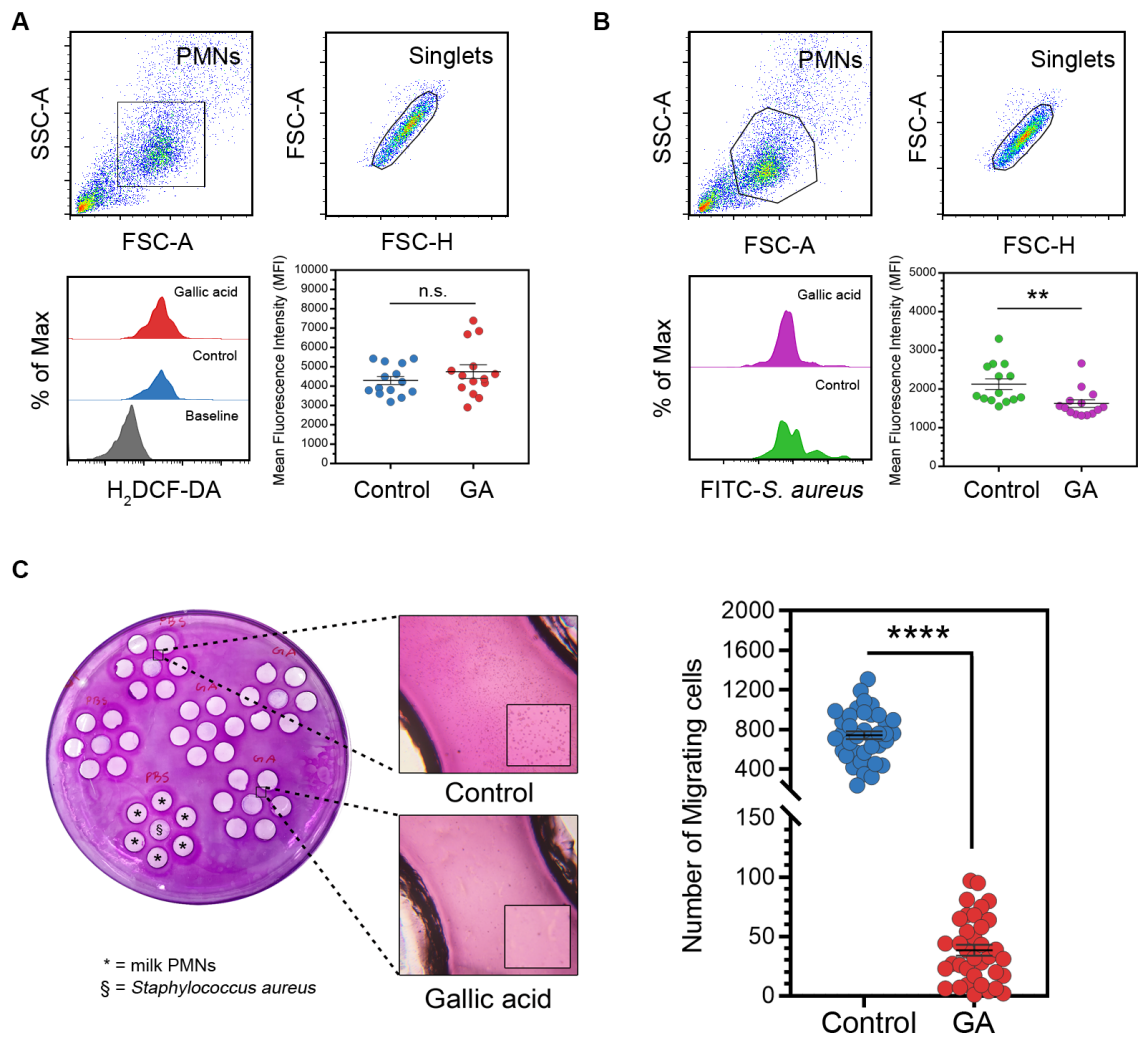
Effects of gallic acid on milk phagocyte ROS production, phagocytosis against *in vitro* challenge of *Staphylococcus aureus*, and migration. **(A)** Gating strategy of milk phagocytes and representative histograms of H_2_DCF-DA (ROS) positive cells (lower left) and scatter dot plots indicated mean fluorescence intensity (MFI) of control or GA-treated cells (lower right). The solid line represents mean ± SEM. Data are from two separate experiments with *n* = 14 data points. Statistical analysis was performed using unpaired Student’s *t* test. **(B)** Gating strategy of milk phagocytes and representative histograms of FITC-*Staphylococcus aureus* positive cells (lower left) and scatter dot plots indicated phagocytosis (MFI) of HBSS treated (control) or GA-treated cells (lower right). The solid line represents mean ± SEM. Data are from two separate experiments with *n* = 14 data points. Statistical analysis was performed using unpaired Student’s *t* test. **(C)** Under-agarose cell migration assay. Milk phagocytes were treated with gallic acid (GA) or HBSS as control and then seeded into the corresponding wells, along with live *Staphylococcus aureus*, as indicated on the plate (left). Representative images of migrating milk phagocytes towards HBSS or bacteria (middle). Scatter dot plots for the number of migrating cells in the control and GA-treated groups (right). The solid line represents mean ± SEM. Data are from two experiments with *n* = 36–39 data points. Statistical analysis was performed using unpaired Student’s *t* test. Statistically significant *p* values are indicated in the graphs as follows: ****p < 0.0001, **p < 0.01 between groups, n.s. not significant.

The purpose of our experiment was to evaluate the influence of gallic acid on the production of intracellular reactive oxygen species (ROS) by employing a fluorescent dye (H_2_DCF-DA) and analyzing it with flow cytometry (Figure 2A). The results from our flow cytometry analysis revealed a slight increase in the amount of ROS production (MFI of 5,307 ± 422.70) in cells exposed to 50 μΜ of gallic acid as compared to HBSS control cells (MFI of 4,885 ± 283.70). However, this difference was statistically non-significant (*p* = 0.41, Figure 2A).

### Assessment of phagocytosis by flow cytometry unveiled that gallic acid hindered the process of internalization and phagocytosis in milk phagocytes

We conducted an *in vitro* study to investigate the effects of gallic acid on the phagocytosis of FITC-labeled *S. aureus* by milk phagocytes. We found that milk phagocytes displayed a comparable phagocytic activity to circulating neutrophils. However, the phagocytic capacity of gallic acid-treated milk phagocytes was lower compared to that of the HBSS-treated control cells (Figure 2B). Upon encountering bacteria, the phagocytic activity of GA-treated cells significantly decreased (MFI of 1,625 ± 98.39) compared to the HBSS control (MFI of 2,127 ± 138.50, *p* = 0.0065, Figure 2B).

### The assessment of milk phagocyte chemotaxis using the under-agarose cell migration assay revealed that gallic acid led to a decrease in cell motility

We proceeded to evaluate the movement of gallic acid-treated milk phagocytes towards live *S. aureus* using an under-agarose cell migration assay (Figure 2C). We observed that the directionally migrating HBSS-treated milk phagocytes were drawn towards the bacteria and were absent in the GA-treated milk phagocytes. The number of migrated cells in the HBSS and GA-treated groups was 741.40 ± 40.11 cells and 38.42 ± 4.58 cells, respectively (Figure 2C). Our results demonstrated that the transmigration of GA-treated cells was significantly different from that of the HBSS control (*p* < 0.0001, Figure 2C).

### The MTT assay and spot dilution assay revealed that gallic acid reduced the effectiveness of milk phagocytes in bacterial killing

**Figure 3:**
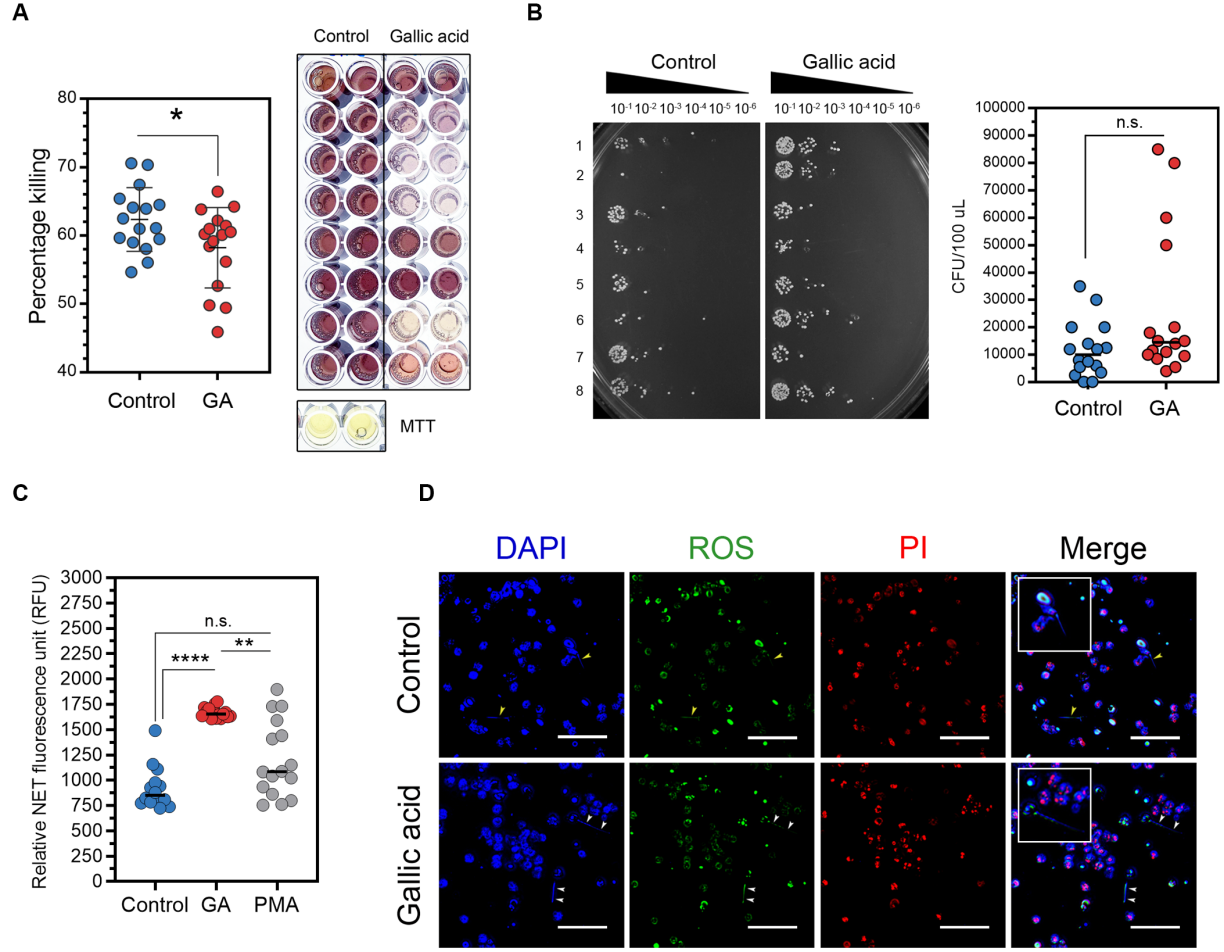
Effects of gallic acid on milk phagocytes *in vitro* bacterial killing and neutrophil extracellular traps (NETs) release. **(A)** Percentage of *Staphylococcus aureus* killing by HBSS- (control) or gallic acid (GA)-treated milk phagocytes by MTT assay. Scatter dot plots indicated the percentage of the bacterial killing of control or GA-treated cells (left). Representative image of the bacterial formazan crystals in 96-well plates by MTT assay (right). The solid line represents mean ± SEM. Data are from two separate experiments with *n* = 16 data points. Statistical analysis was performed using unpaired Student’s *t* test. **(B)** Representative image of serial dilutions of *S. aureus* colonies from MTT assay that were spotted onto plates and incubated overnight (left). Scatter dot plots indicated colony-forming units (CFU/100 μL) of growing *S. aureus* that were recovered from control or GA-treated cells in MTT assay (right). The solid line represents the median. Data are from two separate experiments with *n* = 16 data points. Statistical analysis was performed using the Mann–Whitney *U* test. **(C)** Detection of neutrophil extracellular traps (NETs) in the supernatant (cell-free DNA) by fluorescent plate reader. Scatter dot plots indicated the amount of NET release (RFU) in control, GA-treated, and PMA-treated cells (as pharmacological induction). The solid line represents the median. Data are from two experiments with *n* = 14–16 data points. Statistical analysis was performed using Kruskal-Wallis and Dunn’s multiple comparisons tests. **(D)** Representative images obtained by fluorescence microscopy showing NET structures by milk phagocytes. Cells were stimulated with HBSS (as control) or gallic acid (GA) for 150 min. Extracellular DNA was stained blue, intracellular ROS (green), and cells undergoing necrosis were stained red by propidium iodide (PI). Arrowheads showing NET release into the extracellular space. Insets of merged images are the magnified images of the release of NETs from milk phagocytes. Images were captured at ×100 magnification, scale bar = 50 μm. Statistically significant *p* values are indicated in the graphs as follows: ****p < 0.0001, **p < 0.01, and *p < 0.05 between groups, n.s. not significant.

To evaluate the ability of gallic acid-treated cells to eliminate harmful bacteria in milk phagocytes, we have utilized the MTT assay for bacterial killing. However, our results indicated that live *S. aureus* was phagocytosed and destroyed by milk phagocytes, as shown in Figure 3A. The addition of gallic acid did not significantly enhance the killing of encountering bacteria in this test model, with a percentage killing of 62.37 ± 1.16 in control and 58.23 ± 1.47 in GA-treated cells. GA-treated cells did not exhibit a strong desire to destroy surrounding bacteria. The killing percentage varied significantly among treatments (*p* = 0.0354, Figure 3A).

Investigating bacterial colonies on nutrient agar plates using serial spot dilution assays proved that some bacteria had been eliminated. The experiments showed a significant variation in bacterial colony quantities between control and GA-treated cells. The control group, cells treated with HBSS, had a median of 10,000 CFU/100 μL. In contrast, the experimental group, cells treated using gallic acid, had roughly a median number of 14,500 CFU/100 μL, Figure 3B. Compared to the control group, the number of colonies that were countable in the GA-treated group was higher, implying a reduced killing ability in the GA-treated group (*p* = 0.0917, Figure 3B). On the other hand, our data did not demonstrate a difference in the bacterial killing capacity of milk phagocytes after treatments.

### The quantification of NETs clearly indicated an increase in NET release by milk phagocytes upon treatment with gallic acid

We investigated how milk phagocytes fight extracellular pathogens using NETs. After supplementing cells with HBSS, gallic acid, or PMA, fluorescence plate reader analysis showed that GA-treated cells (1,668 ± 13.85 RFU) and PMA-stimulated cells (1,205 ± 94.06 RFU) released significantly more NETs than HBSS-treated cells (881.10 ± 37.88 RFU, *p* = 0.0002, Figure 3C). Fluorescent microscopy confirmed the presence of extracellular structures (Figure 3D). It was observed that the long NETs extended away from cells irrespective of the substance used. In contrast, non-NET-producing cells displayed intact nuclei, which were visualized using DAPI staining (blue), varying degrees of ROS accumulation (green), and propidium iodide permeation (red). These are typical indicators of damaged, dead, or apoptotic cells, potentially resulting from ROS generation or other stimuli. Figure 3D illustrates the presence of viable NETosis, where cells containing *bona fide* NET were identified by thread-like, extracellular DNA co-localized with ROS.

### Quantitative real-time PCR (qPCR) analysis revealed that gallic acid had an impact on gene expression patterns in milk phagocytes in response to the *S. aureus* challenge

**Figure 4:**
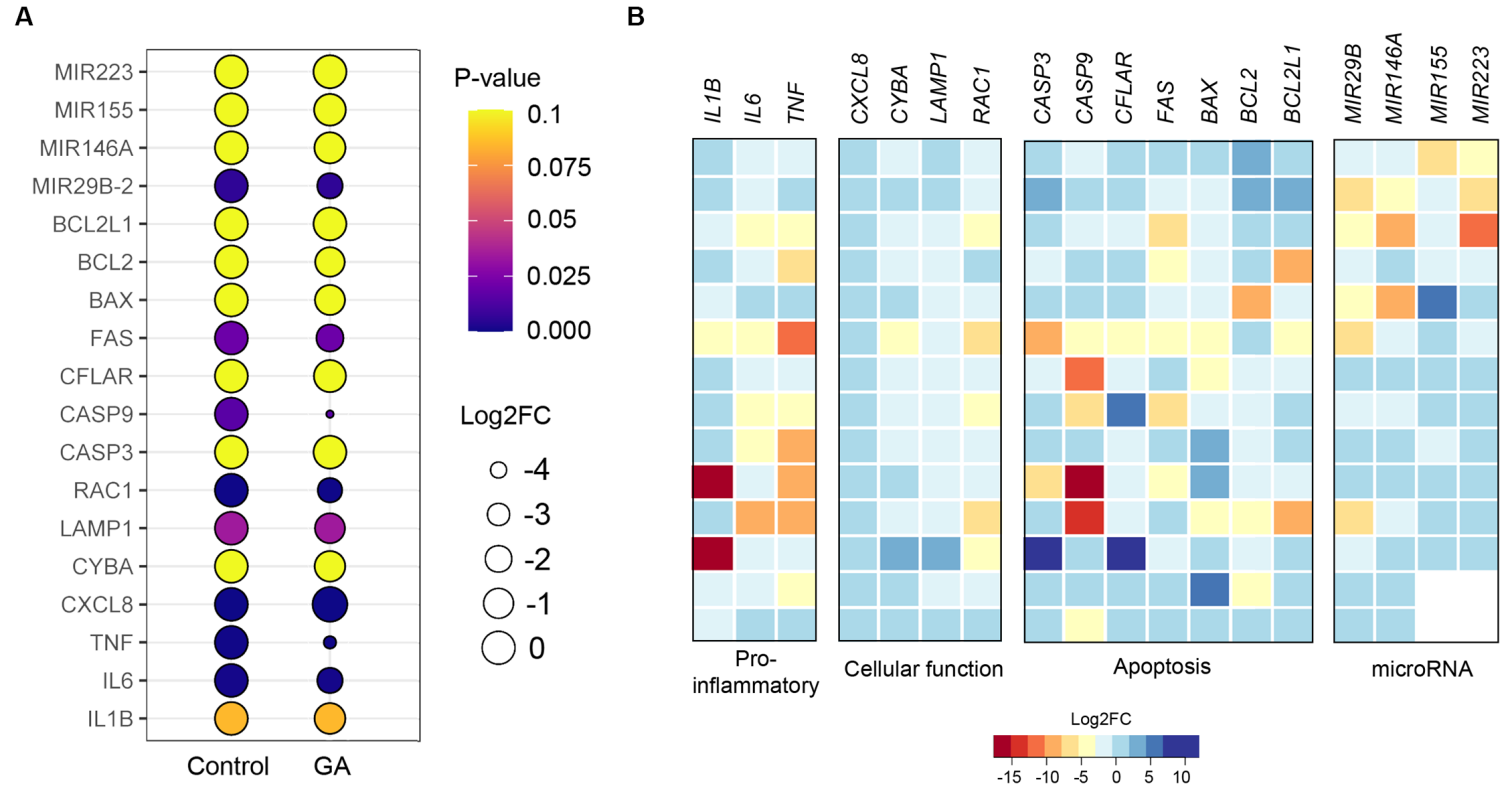
Effect of gallic acid on gene expressions determined by real-time PCR in milk phagocytes. **(A)** The bubble plot shows relative gene expression as log2 fold-change (Log2FC) in HBSS-treated (control) and gallic acid (GA)-treated milk phagocytes. Bubble size is proportional to the mean Log2FC of cells expressing a gene, and color intensity is the expression *p*-value. **(B)** Heatmaps of genes related to pro-inflammatory, cellular function, apoptosis, and inflammatory miRNA. Heatmaps were generated from expression data of GA-treated milk phagocytes, where expression data from control was equal to 0 in Log2FC. Rows represent individual arrays, while columns represent specific genes of interest. A given color scale depicts the Log2FC value for each gene. Data are from two experiments with *n* = 12–14 data points. Statistical analysis was performed using unpaired Student’s *t* test.

We observed changes in gene expression in milk phagocytes with and without gallic acid supplementation. The genes involved in pro-inflammation (*IL1B*, *IL6*, *TNF*), cellular functions: migration, ROS, and phagocytosis (*CXCL8*, *CYBA*, *LAMP1*, *RAC1*), cellular apoptosis (*CASP3*, *CASP9*, *CFLAR*, *FAS*, *BAX*, *BCL2*, *BCL2L1*), and inflammatory microRNAs (*MIR29B*, *MIR146A*, *MIR155*, *MIR223*) were analyzed for the levels of expression between treatments. The expressions of pro-inflammatory genes (*IL1B*, *IL6*, and *TNF*) were reduced in GA-treated milk phagocytes compared to untreated cells (*IL1B*, *p* = 0.08; *IL6*, *p* = 0.001; and *TNF*, *p* = 0.0005; Figure 4A, *IL1B*, *IL6*, and *TNF*). In addition, the migration-related gene *CXCL8* was significantly elevated in the GA-treated group (*p* < 0.0001). The ROS-generating genes and other functional genes (*CYBA*, *LAMP1*, and *RAC1*) were down-regulated by gallic acid activity in milk phagocytes (*CYBA*, *p* = 0.12; *LAMP1*, *p* = 0.03; *RAC1*, *p* = 0.0003, Figure 4A). Pro-apoptotic genes *CASP3* (*p* = 0.97), *CASP9* (*p* = 0.02), *CFLAR* (*p* = 0.64), *FAS* (*p* = 0.02), *BAX* (*p* = 0.23) and anti-apoptotic genes *BCL2* (*p* = 0.17), *BCL2L1* (*p* = 0.96) were expressed at lower levels than controls or unchanged, Figure 4A. In summary, the expression patterns, as mentioned above, are summarized through the creation of a heat map utilizing qPCR data. The heat map displays a log2 fold-change (Log2FC) scale representing the relative mRNA abundance after cellular gallic acid exposure (refer to Figure 4B).

### The western blot analysis demonstrated that gallic acid altered the expression patterns of apoptosis-related proteins in milk phagocytes

**Figure 5:**
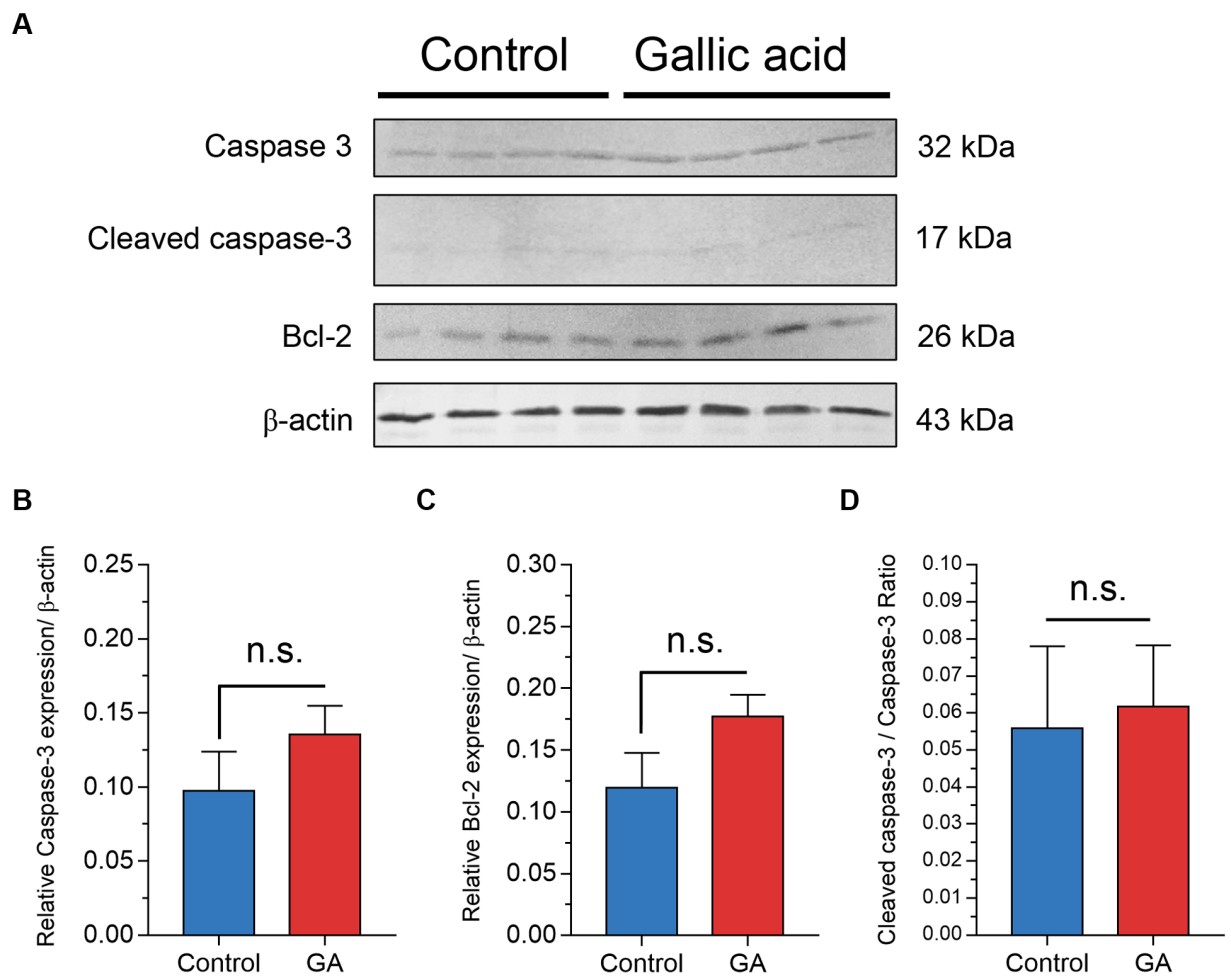
Effect of gallic acid on markers of apoptosis in milk phagocytes by western blot analysis. **(A)** Milk phagocytes were treated with HBSS as control or gallic acid (GA) in the presence of live *Staphylococcus aureus* for 2 h. Western blot analysis of Caspase-3, cleaved caspase-3, Bcl-2, and β-actin as loading control. **(B)** Relative ratio of Caspase-3/β-actin **(C)** Relative ratio of Bcl-2/β-actin **(D)** Cleaved caspase-3/Caspase-3 ratio. Data are from two separate experiments with *n* = 6 data points. Statistical analysis was performed using unpaired Student’s *t* test. Statistically significant *p* values are indicated in the graphs as follows: n.s. not significant.

This report examines whether applying gallic acid treatments leads to cell death, primarily through apoptosis. The fate of the cells after being exposed to gallic acid was examined. Using western blot analysis, we evaluated the expression levels of pro-caspase 3 and cleaved caspase 3 proteins in cell lysates. The results indicated a potential increase in the pro-apoptotic caspase-3 protein (*p* = 0.27, Figures 5A,B) due to gallic acid treatment. However, the presence of cleaved caspase 3, which reflected the extent of cellular apoptosis, did not significantly differ between treatment conditions in this study, Figures 5A–C. These findings regarding caspase 3 expression align with the gene expression analysis (*CASP3*) performed using real-time PCR. The family of anti-apoptotic Bcl-2 proteins, including Bcl-2 itself, is susceptible to the influence of ROS when subjected to oxidative stress conditions. Our findings demonstrated that gallic acid enhanced the expression of the Bcl-2 protein (*p* = 0.11, Figure 5B). Our research also revealed unique insights regarding the expression of the *CYBA* gene, which is involved in ROS regulation. Intriguingly, we observed a considerable down-regulation of this gene (−0.78 ± 0.48), along with a corresponding decrease in the expression of the *BCL2* gene (−1.13 ± 0.80).

## Discussion

Leaves, bark, flowers, fruits, seeds, and nuts are sources of a polyphenolic compound known as gallic acid (25). Numerous studies have proven that gallic acid and its derivatives are crucial for various biological processes (7, 25–27). Moreover, gallic acid has immunostimulant properties that can alter immune responses (25). Gallic acid has been shown to have anti-bacterial properties by disrupting bacterial cell membranes and interfering with enzymatic activities. This was demonstrated by a minimum inhibitory concentration (MIC) of 1,750 μg/mL for *S. aureus* (7). Our results also show that gallic acid can effectively eradicate *S. aureus*, even when administered at a sublethal dose of 1 mM gallic acid in a serial dilution.

Gallic acid has demonstrated safety in cell culture and animal models, as evidenced by many studies (17, 28, 29). In our research, gallic acid exhibited cell viability exceeding 70% when tested on milk phagocytes, thereby establishing the safety level of gallic acid, particularly at a concentration of 50 μM applied to milk phagocytes. The present finding is consistent with the outcomes of research conducted by Yang et al. (30), wherein it was observed that lower concentrations of gallic acid (10, 20, or 50 μM) did not have any impact on the viability of cells. Furthermore, Yen et al. (8) reported that higher concentrations of gallic acid (up to 4.17 mM) were well tolerated in human lymphocytes. Gallic acid is a safe therapeutic agent that has the potential to be used in various medical applications without compromising cell health. This is a notable example of the practical applications of gallic acid.

In addition to its established safety profile, gallic acid has been found to affect cell migration. Research on ascorbic acid, which has similar properties, suggests it may improve chemotaxis (8). In mouse inflammation models, gallic acid decreased total white blood cell count and reduced neutrophil migration (31, 32). Similarly, chlorogenic acid (CGA), a phenolic compound in the same class as gallic acid, significantly reduced the chemotaxis of bovine PMNs when exposed to *S. aureus* (29). Our findings, consistent with previous research, suggest that GA-treated milk phagocytes significantly repelled *S. aureus*, potentially mitigating milk phagocyte migration in the presence of bacteria. Depending on the context, this could initiate inflammation or allow immunological resolution. In some cases, where there is already too much tissue damage, it may be best to prevent phagocytes from reaching the infected site by interfering with cell migration, but further *in vivo* research is required.

This study unveiled that gallic acid displayed pro-oxidative effects through increased intracellular ROS levels. The *in vitro* study has shown that gallic acid has both anti-oxidant and pro-oxidant properties (8). Factors such as the concentration of gallic acid, the presence of metal ions (Fe^2+^ or Cu^2+^), glutathione turnover (11), and the coexistence of other anti-oxidants affect the net anti-oxidant activity of gallic acid (8, 32). It is important to note that gallic acid can also exhibit pro-oxidant behavior due to its susceptibility to oxidation, leading to free radicals. The pro-oxidant properties of gallic acid are typically observed at higher concentrations but act as an anti-oxidant at lower concentrations by effectively scavenging harmful ROS such as superoxide radical (O_2_^−^) and hydroxyl radical (^•^OH). We selected a gallic acid concentration of 50 μM for this study, which is considered relatively high. The results of GA-treated milk phagocytes revealed a slight increase in ROS levels, which is compatible with the current state of the pro-oxidant effects. This would allow us to investigate this effect in more detail.

The evidence suggested that gallic acid may decrease phagocytosis by interfering with superoxide anions, inhibiting myeloperoxidase (MPO) release and activity, and possibly disturbing the assembly of active NADPH-oxidase (18). These complex factors modulate the inflammatory process, affecting phagocytic activity (18). Our investigations have found that gallic acid reduced the phagocytic activity of milk phagocytes against *S. aureus.* Nevertheless, due to limitations, we could not conduct in-depth experiments on NADPH oxidase subunit activity, which is associated with phagocytosis, to explore the mechanism by which gallic acid achieves this effect. The direct bacterial killing property of gallic acid was more effective against gram-positive bacteria, such as *S. aureus,* than gram-negative bacteria *in vitro* (26). Regarding the bacterial killing ability of gallic acid on milk phagocytes against live *S. aureus*, our findings indicated that GA-treated cells did not exhibit significant efficacy in bacterial eradication. Evidence indicated that milk-resident neutrophils could kill *S. aureus*, similar to their blood counterparts. However, their phagocytic and bactericidal activity was lower than that of blood neutrophils (33). This difference might be associated with the rapid production of ROS and free radicals inside the phagosome and phagolysosome during the engulfment of invading bacteria (33). *S. aureus* was correlated with reduced ROS in bovine PMNs, indicating that oxidative pathways played a crucial role in efficiently destroying *S. aureus* (34). The ROS level is just one of several factors that may contribute to our study’s decreased phagocytic and bactericidal activity of milk phagocytes. Although our results indicated a slight increase in ROS levels, it may not be sufficient to guarantee this ability. Additionally, bacterial recognition and adhesion through specific receptors might be involved in this process, but further research is required to understand these mechanisms fully.

In our study, we observed a significant increase in the release of neutrophil extracellular traps (NETs) by milk phagocytes treated with gallic acid. Unlike our study, Haute et al. (19) employed gallic acid treatment followed by LPS exposure to induce apoptosis and NETosis in neutrophils. The authors demonstrated that gallic acid effectively reduced apoptosis and NETosis in neutrophils by inhibiting caspase-3 activation, an essential protease involved in apoptosis (19). Based on published works, primed neutrophils release NETs that could entrap *S. aureus* without killing them (35, 36). The conditions under which the NETs were produced affect the antimicrobial properties, inflammatory response, and host defense against *S. aureus* infection. Due to the constraint in conducting a cell-free NET-killing assay prevents us from concluding that gallic acid promotes NET release in milk phagocytes, resulting in neither immobilizing nor killing *S. aureus*. We believe gallic acid may be a promising treatment for *S. aureus* mastitis, as it promotes NET production by milk phagocytes. However, further research is needed to confirm that NETs can kill extracellular bacteria.

In addition to its impact on neutrophil cellular function, gallic acid has been extensively studied for its anti-inflammatory properties on various inflammatory cell types, including macrophages and lymphocytes. Notably, investigations conducted by Sohrabi et al. (37) have demonstrated gallic acid’s potential to ameliorate inflammation in rats with emphysema by reducing the levels of pro-inflammatory cytokines, namely TNF-α and IL-6. Furthermore, Gong et al. (29) revealed the downregulation of *IL-6*, *IL-8*, and *TLR2* mRNA expression in bovine mammary epithelial cells upon exposure to *S. aureus*, highlighting gallic acid’s capacity to modulate gene expression associated with critical functional processes in neutrophils. These findings underscore the pivotal role of gallic acid in inflammation reduction through its regulatory influence on genes implicated in neutrophil functionality. In gallic acid-treated milk phagocytes, pro-inflammatory genes (*IL1B*, *IL6*, *TNF*) were reduced, consistent with the findings of other researchers (38). The anti-inflammatory effects of gallic acid may be due to its ability to reduce the influx of neutrophils (32). The mechanism behind this anti-inflammatory action involves downregulating nuclear factor-κB (12).

Gallic acid regulates inflammation and modulates the anti-apoptotic process in neutrophils through NF-κB and ERK/MAPK signaling pathways (4, 12, 39). Notably, our results showed that gallic acid treatment increased Bcl-2 levels, which is somewhat consistent with the results reported by Ji et al. (15). On the contrary, gallic acid has been reported to trigger cellular apoptosis through various mechanisms (40). Our study on milk phagocytes revealed caspase-3 activation due to gallic acid treatment, consistent with reports in human neutrophils (19). Regarding cellular longevity, our studies suggest that gallic acid may have potential future applications, particularly in enhancing cell survival during *S. aureus* infection. This is because gallic acid can boost some cellular functions and lifespan, which are vital during infection. However, further research and studies are needed to confirm and validate these findings.

## Conclusion

The viability of milk phagocytes remained unaffected by the substance, suggesting its safe utilization *in vivo*. Our findings suggest that gallic acid has potentially impactful effects on milk phagocytes through cellular and molecular processes. Gallic acid can reduce phagocytosis, bacterial killing, and migration of milk phagocytes while only slightly increasing ROS levels. This could impair their ability to fight infection. In other words, gallic acid appears to have a negative impact on the functions of milk phagocytes, which could potentially disrupt the function of udder immunity. Positively, gallic acid can increase the extracellular release of NETs, which can assist in bacteria entrapment and eradication. Nonetheless, this process could also damage adjacent tissues. As expected, gallic acid treatment altered gene expression patterns in milk phagocytes, particularly in the genes involved in inflammation and apoptosis, in response to *S. aureus*. These effects have a number of implications for the potential use of gallic acid in manipulating the udder immune system and treating udder inflammation. Regardless of these reports and discoveries, it is important to note that our *in vitro* findings may not fully reflect the intricacy of gallic acid’s impacts on biological relevance *in vivo*. Therefore, it is necessary to conduct further research using *in vivo* models or animal studies to gain a complete understanding and provide valuable insights into the therapeutic potential of gallic acid.

## Data availability statement

The raw data supporting the conclusions of this article will be made available by the authors, without undue reservation.

## Ethics statement

The animal studies were approved by Animal Care and Use Committee (FVM-ACUC), Ref. No. S27/2564, at the Faculty of Veterinary Medicine, Chiang Mai University. The studies were conducted in accordance with the local legislation and institutional requirements. Written informed consent was obtained from the owners for the participation of their animals in this study.

## Author contributions

RM: Conceptualization, Investigation, Methodology, Resources, Writing – original draft, Writing – review & editing. PC: Conceptualization, Investigation, Methodology, Resources, Writing – original draft, Writing – review & editing, Formal analysis, Funding acquisition, Validation, Visualization. DN: Investigation, Writing – review & editing. TK: Investigation, Writing – review & editing. AS: Investigation, Resources, Writing – review & editing. WS: Funding acquisition, Resources, Writing – review & editing.

## Funding

This work was supported, in part, by grants from the Fundamental Fund (FF2565), Thailand Science Research and Innovation (TSRI), 2022, under Grant No. FRB650031/0162 through Chiang Mai University and the Research Center of Producing and Development of Products and Innovations for Animal Health and Production, Chiang Mai University, Chiang Mai, Thailand (Grant No. R000029886).

## Conflict of interest

The authors declare that the research was conducted in the absence of any commercial or financial relationships that could be construed as a potential conflict of interest.

## Publisher’s note

All claims expressed in this article are solely those of the authors and do not necessarily represent those of their affiliated organizations, or those of the publisher, the editors and the reviewers. Any product that may be evaluated in this article, or claim that may be made by its manufacturer, is not guaranteed or endorsed by the publisher.
